# A gender synchronized family planning intervention for married couples in rural India: study protocol for the CHARM2 cluster randomized controlled trial evaluation

**DOI:** 10.1186/s12978-019-0744-3

**Published:** 2019-06-25

**Authors:** Anvita Dixit, Sarah Averbach, Jennifer Yore, Gennifer Kully, Mohan Ghule, Madhusudana Battala, Shahina Begum, Nicole E. Johns, Florin Vaida, Prashant Bharadwaj, Natalie Wyss, Niranjan Saggurti, Jay Silverman, Anita Raj

**Affiliations:** 1 Joint Doctoral Program in Public Health (Global Health track), University of California San Diego/San Diego State University, San Diego, USA; 2 Center on Gender Equity and Health, School of Medicine, University of California San Diego, 9500 Gilman Drive #0507, La Jolla, San Diego, CA 92093-0507 USA; 3 Department of Obstetrics, Gynecology and Reproductive Sciences, School of Medicine, University of California San Diego, 9300 Campus Point Drive, La Jolla, San Diego, CA 92037 USA; 4 Population Council, Zone 5A, Ground Floor, India Habitat Center, Lodi Road, New Delhi, 110003 India; 5 Department of Biostatistics, ICMR-National Institute for Research in Reproductive Health, J.M Street, Parel, Mumbai, 400012 India; 6 Division of Biostatistics, Department of Family Medicine and Public Health, University of California San Diego, 9500 Gilman Drive #0507 La Jolla, San Diego, CA 92093-0507 USA; 7 Department of Economics, Division of Social Sciences, University of California San Diego, 9500 Gilman Drive #0507 La Jolla, San Diego, CA 92093-0507 USA; 8 Department of Education Studies, Division of Social Sciences, University of California San Diego, San Diego, USA

**Keywords:** Family planning, Cluster randomized controlled trial, Intervention, Contraceptive use, Gender equity, Marital sexual violence

## Abstract

**Background:**

Prior research from India demonstrates a need for family planning counseling that engages both women and men, offers complete family planning method mix, and focuses on gender equity and reduces marital sexual violence (MSV) to promote modern contraceptive use. Effectiveness of the three-session (two male-only sessions and one couple session) Counseling Husbands to Achieve Reproductive Health and Marital Equity (CHARM) intervention, which used male health providers to engage and counsel husbands on gender equity and family planning (GE + FP), was demonstrated by increased pill and condom use and a reduction in MSV. However, the intervention had limited reach to women and was therefore unable to expand access to highly effective long acting reversible contraceptives such as the intrauterine device (IUD). We developed a second iteration of the intervention, CHARM2, which retains the three sessions from the original CHARM but adds female provider- delivered counseling to women and offers a broader array of contraceptives including IUDs. This protocol describes the evaluation of CHARM2 in rural Maharashtra.

**Methods:**

A two-arm cluster randomized controlled trial will evaluate CHARM2, a gender synchronized GE + FP intervention. Eligible married couples (*n* = 1200) will be enrolled across 20 clusters in rural Maharashtra, India. Health providers will be gender-matched to deliver two GE + FP sessions to the married couples in parallel, and then a final session will be delivered to the couple together. We will conduct surveys on demographics as well as GE and FP indicators at baseline, 9-month, and 18-month follow-ups with both men and women, and pregnancy tests at each time point from women. In-depth interviews will be conducted with a subsample of couples (*n* = 50) and providers (*n* = 20). We will conduct several implementation and monitoring activities for purposes of assuring fidelity to intervention design and quality of implementation, including recruitment and tracking logs, provider evaluation forms, session observation forms, and participant satisfaction surveys.

**Discussion:**

We will complete the recruitment of participants and collection of baseline data by July 2019. Findings from this work will offer important insight for the expansion of the national family planning program and improving quality of care for India and family planning interventions globally.

**Trial registration:**

ClinicalTrial.gov, NCT03514914.

## Plain English summary

The protocol aims to describe the implementation and evaluation of a gender synchronized family planning intervention CHARM2 [Counseling Husbands and wives to Achieve Reproductive health and Marital equity 2] to wives ages 18–29 years and their husbands through local health providers in Maharashtra, India. A gender matched healthcare provider will conduct five sessions with the couple including, two sessions with husbands, two sessions with wives, and one with the couple together. The intervention aims to improve contraceptive use as well as reduce unintended pregnancy and marital sexual violence. The participants will also be offered expanded contraceptive method choices. CHARM2 not only engages husbands, but also focuses on women’s reproductive autonomy and improved availability of LARCs. The intervention also engages public as well as private health care providers to support infrastructure for implementation. The study will be evaluated using quantitative surveys at baseline, 9 months and 18 months follow-ups, and qualitative in-depth interviews with couples and health care providers as well. The findings from this study will inform the national public family planning program in India and offer insights for interventions globally.

## Background

Globally, 41% of all pregnancies are unintended [1, 2], increasing risk for poor maternal and neonatal health outcomes [3–5]. Most unintended pregnancies occur among women who use no contraception; one in three is due to contraceptive failure [6]. Poor, rural and young women are at greatest risk for both contraceptive non-use and failure, with the latter being a consequence of reliance on traditional and short-acting contraceptives (e.g., pill, condom) rather than more effective long-acting reversible contraceptives (LARC) (e.g., IUD) [5, 7]. These young women are also more vulnerable to marital sexual violence (MSV), compromising their reproductive control and contraceptive use [8, 9]. Family planning interventions that engage men as well as women, address gender inequities in marital relationships, support women’s reproductive control and safety from MSV, and provide the full range of available effective contraceptive options including LARC are needed. Rural India, with some of the lowest rates of contraception and highest rates of marital violence globally [10–12], is an important context in which to implement and test such interventions.

The Counseling Husbands and wives to Achieve Reproductive health and Marital equity 2 (CHARM2) intervention is based on findings from our evaluation of the original CHARM study, an innovative, brief (2 men’s sessions and 1 couples’ session), gender equity and family planning (GE + FP) intervention delivered by male health providers in rural Maharashtra, India. A two-arm cluster randomized controlled trial (RCT) compared the CHARM intervention to the standard of care (SOC) public health services (community outreach to women and linkage to family planning (FP) clinics) to determine its impact on reversible contraceptive use and unintended pregnancy [13]. CHARM demonstrated increased modern contraceptive use, but no reduction in unintended pregnancy [14]. This male-delivered model had strong participation from men (91%), but less from couples (51%), largely due to preferences for gender-matched providers [14]. Poor reach to women and provision of only moderately effective contraceptives (pill, condom) [5], likely compromised unintended pregnancy outcomes. CHARM was associated with a reduced risk for MSV, a concern for almost 1/3 of women in the study sample [14] and a significant risk factor for condom non-use and oral contraceptive failure [15, 16]. These findings indicated that original CHARM sessions for men would benefit from sessions for women, delivered by a female auxiliary nurse midwife (ANM) and inclusive of a broader array of contraceptive options, including LARC. The recent expansion of FP services in India to prioritize nurse-delivered IUDs [17] and more recently, injectables [18], is aligned with study goals and will be supported by implementation.

The CHARM male engagement focused FP intervention required adaptations to better support women’s engagement and reproductive autonomy. These include improved availability of LARCs, and female-focused gender equity and FP counselling sessions delivered by female providers, paired with the male provider-delivered men’s sessions. We have incorporated these adaptations into the CHARM2 intervention, which we are evaluating via a two-arm cluster RCT. The CHARM2 intervention further engages public-private partnership to support infrastructure for implementation, if successful. This paper describes our study protocols for the quantitative, qualitative, and implementation-science based evaluation of CHARM2 in rural Maharashtra, India. Quantitative evaluation will assess the impact of CHARM2 on contraceptive use, unintended pregnancy, and MSV. We will also describe elements of our implementation framework and monitoring to ensure high-quality delivery of the intervention and for purposes of its replication, as well as a qualitative evaluation of the intervention in terms of participant and provider perceptions of the mechanisms that may explain behavior change.

## Methods

The study has two phases. Phase 1 was preparatory and entailed mapping the project area and preparing communities for the planned research. Phase 2 entails implementation and evaluation of CHARM2, using a two-arm cluster RCT in the geographic clusters mapped in Phase 1, via surveys and pregnancy testing at baseline and 9 & 18-month follow-up with *n* = 1200 couples, coupled with in-depth interviews (IDIs) with providers, couples, and stakeholders.

### Setting

This study is being conducted in Pune District, Maharashtra, which has a rural population of 3.7 million residents across 2000 villages [19, 20]. In rural Pune, female illiteracy is 27%, and the child sex ratio is 833 girls per 1000 boys (indicative of son preference/missing girls) [19, 20]. Only 25% of non-sterilized women of childbearing age use modern contraception [21]. Within Pune District, we focus on rural Junnar Taluka (taluka: geographic sub-district area), which is comprised of 183 villages (pop. 399,000). As in most of India, Junnar’s public health structure is hierarchical, with two community health centers (CHCs) offering inpatient and surgical facilities and 12 primary health centers (PHCs; 6 PHCs under each CHC) with maternal delivery beds, infant incubators, and FP services. Under each PHC, there are 3–10 sub-centers (SCs; total 74 in Junnar), in which ANMs provide maternal, newborn, and child health (MNCH) and family planning services. Each SC serves a population of about 5000 in non-tribal areas and 3000 in hilly areas. In consultation with the department of health and family welfare of Maharashtra state and Pune district health offices, 5 PHCs in Junnar taluka were selected for the CHARM2 study. These five PHCs includes 20 subcenters, providing health services to approximately 150,000 individuals. There are nearly 30,000 households spread across 41 villages and hamlets (habitant areas attached to administrative demarcation of a revenue village).

To understand the study area, our field research team conducted a detailed community mapping exercise under the supervision of the leadership team in India. We used maps generated by PHCs to identify SC boundaries and major villages within SCs. We collected the following information at the community level: key persons (ANM, accredited social health activist (ASHA), integrated child development services (ICDS) worker, and heads of village panchayats: a village council), availability of infrastructure (SC, ICDS Anganwadi center and village panchayat office), transportation facilities (road, rail network), topographic features (hilly area), hamlets and villages, distance to the nearest PHC, and locations of private health providers. In addition, we reviewed seasonal climatic conditions such as rainfall and temperatures to understand the feasibility of work by month. Further, population characteristics, work force, schooling, employment opportunities, and in and out migration were collected. A physical map of the study area was developed to depict the key information collected. These data help clarify cluster geographies for randomization.

### CHARM2 intervention

#### Theoretical framework

As with the original CHARM intervention, the CHARM2 intervention is based on Bandura’s Social Cognitive Theory (SCT) [22] and Theory of Gender and Power (TGP) [23]. SCT has been identified as one of the most commonly used behavioral theories in FP interventions [24]. SCT posits that behavior change, in this case contraceptive use, is more likely if an individual has positive outcome expectations for engaging in the behavior (e.g., believes that birth spacing will produce healthier children), feels capable of engaging in and controlling the behavior (i.e., self-efficacy to use contraception), and has an environment supportive of the behavior (e.g., access to FP services). Hence, SCT would support the use of FP education and skills building together with improved contraceptive access. TGP is a social-structural theory that posits that the gender-based power dynamics inherent to many heterosexual couples are due to societally reinforced social norms [23]. Such norms can facilitate male control over sexual and reproductive decision-making, including contraceptive use. Some men may even use violence, including MSV, to control their female partners. Hence, counseling that can affect normative beliefs around gender equitable family planning, particularly if the counseling is delivered by a respected and gender-matched role model (local health providers and counselors; ideally inclusive of a public health nurse for delivery of women’s sessions), can be useful in improving contraceptive use in the context of safer and healthier relationships.

#### Development of CHARM2

We developed the CHARM2 curriculum using the original CHARM curriculum [14], updating and adding information based on the Government of India’s family planning resources including standard public health counseling guides and details on available contraceptives [25–28]. CHARM2 differs from the original CHARM intervention by adding two sessions just for wives, delivered by a female provider or counselor trained in family planning, and including a broader family planning method mix including IUD. For both the men’s and women’s sessions, we again used Government of India resources, but also added an adapted version of Population Council’s Balanced Counseling Strategy (BCS) tool, to support patient-centered contraceptive choice through information exchange that allows for selection of a method that meets the reproductive needs of the client [29]. Additionally, the women’s session included counseling on reproductive coercion, to guide women to consider how they may safely engage in contraceptive use if they choose and a partner is opposed to it and/or abusive [30].

Once there was clarity on the model and approach, a team led by a family planning clinical researcher developed a detailed curriculum, created a flip chart for use as a tool for CHARM2 providers to use with participants in the field, and generated a set of index cards detailing use, duration of action, efficacy, side effects and contra-indications for each method. The team also developed a training manual on how to use the curriculum as a reference guide for providers. The curriculum included extensive details on contraceptives to ensure safe and up-to-date provision by providers. The curriculum and flipchart detailed Women’s Sessions 1 and 2, Men’s Sessions 1 and 2, and the Couples’ Session. A graphic artist then worked with the team to update or create additional graphics for all materials. Once the materials were developed, local family planning providers reviewed and provided feedback, which we revised and finalized in order to ensure user-friendly materials. We used these materials for the training of CHARM2 providers, as well as for program implementation.

Through provider training and the curriculum, emphasis on the right to respectful and non-coercive family planning counseling was included as part of the CHARM2 approach [31]. The curriculum was designed to support providers to exchange information with participants, to allow them to choose a method that suits their reproductive goals. Additionally, providers delivering the sessions are local trained health providers and counselors, including for the women, qualified auxiliary nurse midwives (ANMs) within the public health system. The ANM’s training and position facilitates access to publicly available contraceptives and allows her to provide IUDs to women who opt for them free of charge, though all CHARM2 providers are given access to publicly funded contraceptives as part of their participation in this study, as we have connected the study to the local district health centers and primary health centers. While injectables were not available through the Maharashtrian public health system, if they become available through the course of the trial, ANMs would also be in a position to provide that option. As noted previously, private female providers can deliver the intervention in the absence of any available ANM, which can be the case for subcenters with vacancies.

#### Overview of the CHARM2 intervention

As noted above, the CHARM2 intervention involves five gender, culture and contextually-tailored FP + GE counseling sessions. (Table 1) Gender-synchronized sessions are delivered individually to husbands by a trained male health provider (2 sessions), and in parallel, delivered individually to wives by a trained female provider (2 sessions). Following the delivery of the individual sessions, there is a couple counseling session (1 session), delivered by either the male or female provider who delivered the individual sessions, whichever is available. We anticipate that these will often be delivered by female providers, who may be more available to this project than male providers. Session content and length are noted in Table 1 below. Sessions are provided in a confidential setting including at the community center or within the clinical setting. For each couple, all three sessions are to be delivered across 4 months with 1 month between the sessions. The men’s first session must be delivered before or at the same time as the female session. We provide all sessions and contraceptives at no cost to participants. Wives, in-laws and other decision-makers may be included in men’s session two if requested by the male participant. Given concerns about IUD side effects [32] such as cramping [33], as well as findings of high reversible contraceptive failure in India [11], the female provider will provide follow-up on contraceptives to support contraceptive continuation and satisfaction [34].

**Table 1:** CHARM2 Intervention Program Outline for Rural Young Couples

SESSION 1	
For Men (20–40 min); Delivered by Male Health Provider • Assess client’s FP knowledge and fertility goals; provide an overview of FP types/effectiveness/availability, encouraging discourse around methods that suit his needs. • Provide info on maternal and child health benefits of FP, as well as delayed first childbirth • Assess sex risk of man: extramarital sex; provide basic HIV/STI prevention information • Briefly assess if a man has discussed FP with his wife; assess & encourage joint FP decision-making • Highlight the importance of male involvement in FP • GE issues- son preference • Review again client’s FP goals; encourage consideration of contraception. For Women (20–40 min); Delivered by ANM • Assess client’s FP knowledge and fertility goals; provide an overview of FP types/effectiveness/availability, encouraging discourse around methods that suit her needs. • Provide info on maternal and child health benefits of FP, as well as delayed first childbirth • Assess sex risk of woman: extramarital sex; provide basic HIV/STI prevention information • Briefly assess if a woman has discussed FP with her husband; assess & encourage joint FP decision-making • Highlight the importance of male involvement in FP • GE issues- son preference, sexual/reproductive control/coercion • Review again client’s FP goals; encourage consideration of contraception.	
SESSION 2	
For Men (20 min); Delivered by Male Health Provider • Assess client’s FP goals; review FP types/effectiveness to support these goals, encouraging discourse around methods that suit his needs. • Review previously identified barriers to FP uptake- such as the desire for sons or pressure from in-laws • Assess if a man has discussed FP with his wife; practice how to communicate about FP • Assess marital violence and sexual communication; reinforce non-use of violence and respectful communication; encourage joint FP decision making with wife • Highlight the importance of male involvement in FP, affection in a sexual relationship • GE issues- son preference, sexual/reproductive control/coercion • Review again client’s FP goals; encourage consideration of contraception. For Women (20 min); Delivered by ANM • Assess client’s FP goals; review FP types/effectiveness to support these goals, encouraging discourse around methods that suit her needs. • Review previously identified barriers to FP uptake- the desire for sons or pressure from in-laws • Assess if the woman has discussed FP her husband; practice how to communicate about FP with husband • Discuss marital violence and sexual communication; reinforce non-use of violence and respectful communication; encourage joint FP decision making with husband • Highlight the importance of male involvement in FP • GE issues- son preference, sexual/reproductive control/coercion • Review again client’s FP goals; encourage consideration of contraception	
SESSION 3- For Couples Together (20–40 min); Delivered by ANM or Male Provider • Provide an overview of health benefits and types/effectiveness of FP, including LARC methods; Assess FP goals; Counsel on fertility and FP joint decision-making; Validate Contraceptive Choices^a^ • GE issues- son preference • Discuss marital communication, sexual communication- FP and MSV • Provide support to obtain methods; follow-up with women regarding satisfaction with contraception every 2 months until satisfaction and safety are confirmed	

^**a**^NOTE: This piece of the CHARM2 ANM session is SOC for ANM FP services

### CHARM2 provider selection and training

#### Provider selection

CHARM2 engages both female (ANM) and male health providers. All ANMs operating in an intervention SC at the time of intervention delivery were selected for inclusion in this study. Per government policy, all ANMs are trained in family planning counseling and services including IUD insertion. To identify ANMs, we collected a list of ANMs working across all SCs within participating PHCs. We approached PHCs in our study site and gained approval for collaboration on this project, in preparation for the study. We compiled contact details for the ANMs and their SCs and approached ANMs from the intervention clusters (10 SCs) to confirm willingness and ability to conduct counseling sessions with female intervention participants for individual sessions and with husband and wife together for the couple session. In that process, we learned that ANM vacancies at SCs are not uncommon, and some ANMs were not yet trained in IUD insertion. In cases of vacancies, ANMs at nearby SCs cover the SC with a vacancy. This finding complicated the original design, and we addressed this concern through the following means: We provided all ANMs in intervention SCs, regardless of whether they received IUD insertion training, the CHARM2 training, but training and data collection were prioritized in SCs with no vacancies, to allow time for the vacancy to be filled. Local female providers not at SCs were also included as back-up in cases where ANMs were unavailable.

To select the male providers, we identified local male allopathic and non-allopathic (AYUSH, traditional medicine) providers working in our intervention SCs via lists maintained by PHCs and through the mapping procedure we undertook in preparation for the study. These were largely private providers. Study staff approached all providers with allopathic or AYUSH credentials to assess their willingness to participate in the study. An additional criterion for selection was no plan to move from the local area in the next 2 years, to ensure continuity of care. We ultimately decided to include both male and female allopathic and AYUSH providers in our selection process, as female providers can serve as CHARM2 female participants in cases where ANMs are not available due to vacancies, as noted above. As back up in cases of shortages of male providers, we also included and trained male social workers and male multi-purpose health workers (MPWs) from the public health system to deliver the CHARM2 intervention.

#### Provider training

We trained all willing and eligible providers to participate in the CHARM2 intervention. In our initial 2-day training, we trained a total of 39 providers, including 8 male and 20 female private providers, and 12 male and 9 female providers working in the public health system. We provided an initial training from behavioral and public health scientists. The training included an introduction to the objectives and design of the CHARM2 intervention program, and why we designed it, as well as background on the original CHARM intervention. We then provided a review of contraceptives and family planning counseling approaches, and education on the value of healthy pregnancy spacing, high-quality interpersonal communication between husband and wife and between patient and provider, and legality and safety of abortion. We then held facilitated discussions on social and gender norm-related barriers to family planning use, including underlying values of gender equity and reproductive choice, shared family planning decision making for couples, fertility norms and expectations, son preference, decision-making control, respectful marital communication, masculinity norms, in-law involvement, spousal violence, and reproductive coercion from husbands and in-laws. Post-test evaluations of this training indicate good clarity across providers on issues of family planning and gender equity.

We expect to provide more training sessions given the turnover of staff at SCs and PHCs as well as some anticipated loss of other private providers. We also anticipate refresher trainings for trained providers immediately prior to roll out of an intervention in their SC. The booster trainings will focus on family planning options, non-coercive and high-quality delivery of care, and social and gender norms constraining family planning practices. Additionally, as ANMs may be serving more than one SC, there may be a risk for contamination if she is in both an intervention SC and a control SC; in these cases, we will provide training on ways to avoid contamination. (See Randomization section below.)

We will track training of all providers and monitor intervention delivery to support improved quality of implementation among providers where needed. We will also maintain data on providers in terms of their sex, educational qualifications, years of experience in practice, years of experience delivering FP counseling, patient load, and hours/days of availability to participate in CHARM2. These data will allow us to assess potential biases in the retention of providers in the study.

### Outcome evaluation of CHARM2

#### Study design

**Fig. 1:**
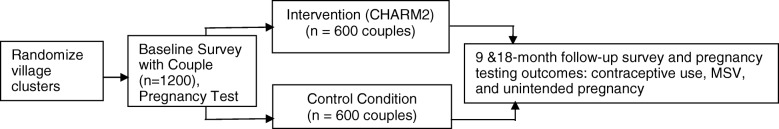
Outcome Evaluation Study Design

A two-arm cluster RCT will compare the intervention arm (FP + GE delivered over 5 sessions with men, women, and couples) with the control arm receiving SOC FP services from the public health system. Surveys are administered to all participants (intervention and control arms), and pregnancy tests from all female participants, at baseline, 9-month follow-up and 18-month follow-up We will evaluate the impact of CHARM2, relative to the control condition, on reversible contraceptive use, pregnancy, and MSV over time. (See Fig. 1.)

#### Randomization

To maintain the CHARM2 intervention within the public health system and utilize the system’s trained ANMs to deliver the intervention sessions to women, we randomized at the SC level, i.e., the public health system’s most local facility, at which ANMs are located. Hence, SCs operated as our cluster for this RCT. Given that SCs are under PHCs, as noted above, we randomized within PHCs, to ensure that administrative cultures of given PHCs did not create bias in our design. We then randomized our 20 SCs (i.e., our clusters), stratified within our three PHCs, into intervention and control conditions in the month prior to enrollment initiation. Research staff are not blinded to the treatment condition assignment to clusters.

#### Recruitment

Within each SC, we randomly selected households with couples meeting inclusion criteria into this study. Our field research staff, in male-female pairs, conduct household screening to identify eligible couples, and then select *n* = 60 couples on the list, per SC, for study inclusion. In a small cluster if the total number of couples is less than or equal to 60, all couples are recruited. In a more populous cluster if the couple size is more than 60, we randomly select 60 couples from the list. In geographically large clusters, we limit household screenings to most populous areas to reduce burden on the staff for recruitment. If an eligible household has two or more couples meeting eligibility criteria, a Kish Grid is used to determine the couple to be selected. All participants provide informed consent before screening and are provided with information about local available publicly supported family planning services.

Eligible study participants include couples in which women report to be between 18-29 years of age and the couple is not sterilized, has resided in the village together for 3 months, is planning to stay in the current place of residence for at least 2 years, is fluent is Maharti and are both willing to participate in the study. Ineligible participants include any couple that identifies as having a spouse with cognitive impairment and/or as sterile/infertile. Pregnant women are eligible to participate, as study outcomes, including contraceptive use and unintended pregnancy, are still possible and meaningful at follow-up for those pregnant at baseline. For those that decline study participation, the reason for refusal is noted, and demographics collected for consideration of participation bias.

#### Study procedures

Once we select couples for study inclusion from the screened household listing of eligible couples per cluster, research teams visit the households of these couples to invite them into the study. Staff make at least three attempts to enroll selected couples into the study. Eligible couples complete the written informed consent process immediately prior to survey participation. Research staff conduct surveys using electronic tablets with couples, separately and with gender-matched interviewers, in a private or confidential setting. We use electronic tablets rather than paper surveys to reduce the likelihood of interviewer error and loss of data, and to eliminate secondary data entry efforts for quality control.

Following completion of the women’s survey, female participants take a urine pregnancy test under the direction of the female research staff member in a private location, away from the husband. The husband is not informed of the pregnancy test or results. The pregnancy test is not carried out if the woman is < 40 days postpartum, 30 days post miscarriage, or currently pregnant in the 2nd/3rd trimester. A woman may refuse to test for any reason. Test results are available within three minutes of testing and staff add test results into the survey. The research investigators wait a minimum of two full minutes before looking at the pregnancy test result, and then collect and dispose of it. If a woman asks for further information or confirmation on whether she is pregnant, the enumerator must direct her to contact the SC/PHC. Enumerators record all reasons for pregnancy test refusal. The interviewer then notifies the woman of her test results and offers information about her local SC, PHC and contact details of an ASHA to seek antenatal and family planning services as appropriate. Staff keep the pregnancy tests and dispose of them at the end of the day, to ensure proper and confidential disposal.

The female research staff informs all women, regardless of treatment group, of available family planning services in the area. If domestic violence is identified, the research staff also provides contact information for Mahila Hakka Suraksha Samiti, Nashik, a Pune-based domestic violence program that can provide safety and protection for women. If a woman indicates immediate life-threatening abuse, she is withdrawn from the study and provided the appropriate referrals. Staff do not inform husbands about these referrals or the violence questions. All participants are informed that free contraceptives are available through the public health system, domestic violence is illegal, and abortion is legal.

After the baseline survey, research staff inform intervention couples regarding locations and contacts for delivery of the CHARM2 intervention sessions and support and link them to their CHARM2 provider, with assistance from ASHA operating at the village level. Participants are reminded that data will be collected from them again in 9 & 18 months. Research team members further assist couples to make their appointments with the providers for intervention sessions. Following appointment confirmation, research staff contact participants 3–4 days prior to the appointment and the day before, to further support the initial visit. Provider visits can occur at a location of the participant and provider’s choosing, such as the clinic or in the participants’ community, as long as privacy for counseling is possible. Research staff provide control group participants with SOC information regarding clinic locations and FP services available at the SC and PHC. We also inform ASHAs of these participants so they may support participant linkage to the public health system.

Research staff, ideally the same interviewers who collect data at baseline, will conduct follow-up assessments at 9- & 18-months post-baseline with both treatment and SOC groups using the same procedures as in baseline data collection. Study participants do not receive incentives. Please see Table 2 for the schedule of enrollment, intervention, and assessments.

**Table 2:**
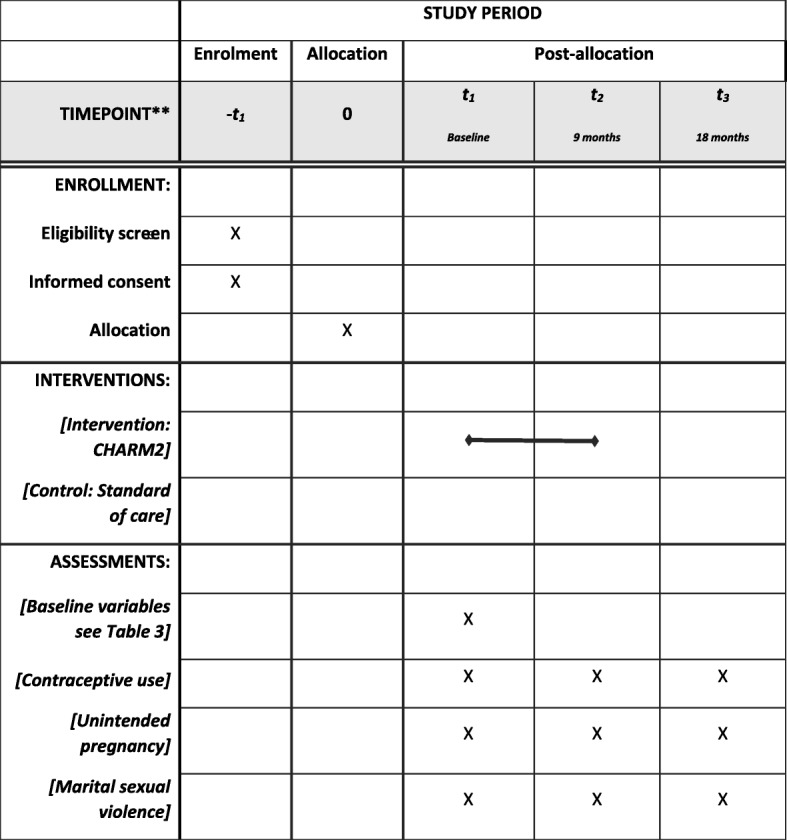
Schedule of enrollment, intervention, and assessments

#### Outcome measures

Baseline and follow-up surveys were created by the US and India-based CHARM2 team, using validated measures from India’s Demographic Health Survey (DHS-NFHS-4) and original CHARM study survey wherever possible. For additional measures, we sought those previously published in peer-reviewed papers, demonstrating good psychometric properties, and previously used in India. The survey includes items and scales on demographics, marital history, control over resources, pregnancy and fertility histories and preferences, family planning history and counseling experiences, gender role norms, marital violence and relationship dynamics, sexual history, and mental health. (See Table 3 for more details on constructs measured. Survey and measurement citations are available upon request).

**Table 3:** Content of CHARM2 Survey

Topics and Constructs	Description
Socio-economic and demographic characteristics	Age, residency, education, religion, caste, household members, employment, income, economic assess ownership, food insecurity, debt, proximity to parents, tobacco and substance use
Marital factors	Number of married years, age at marriage, marital choice, consanguinity in marriage
Control over resources	Household decision making, economic decision making, fertility, and contraceptive decision making
Fertility history and fertility intention	Pregnancy history, menstruation, hygienic practices, delivery, post-natal care, unwanted pregnancy, postpartum FP, male involvement in ANC, fertility desire preferences, STI/STD symptoms
Family planning	Knowledge of FP, source of information, ever use, current use, barriers to use, FP discontinuation, intention to use, contraceptive communication, abortion, knowledge, and resources about FP in community, contraceptive self-efficacy, attitude towards home IUD insertion
Fertility attitudes	Fertility desire, contraceptive preferences, fertility preference, contraceptive decision making, attitudes towards FP use, attitude towards FP reproductive coercion, attitude towards husband involvement in FP, provider FP coercion, interpersonal quality of FP services
Male gender norms (assessed only with men)	Attitude towards gender roles
Gender-based abuse/control (assessed only with women)	IPV-physical, verbal and sexual, injuries, help-seeking, attitude towards physical abuse, witnessing parental IPV, reproductive coercion, mobility, public safety,
Sexual risk behaviors and STI symptoms	Number of partners, commercial sex use, STI and UTI symptoms
Mental health	Depression, generalized anxiety and suicidal ideation
Marital relationship quality	Marital agreement, happiness and future success of relationship
Pregnancy	Pregnancy test

NOTE: Details on scales and measures used for this survey can be made available upon request

Subsequent to development of the survey, multiple English/Marathi bilingual survey researchers trained and experienced in working on sexual health and family planning studies reviewed the survey for clarity, accuracy, and ease of use. Trained field investigators then pilot tested the survey tool using paper surveys with 20 eligible couples from a single village outside of the study area. Pilot testing allowed for the collection of feedback regarding question administration, wording, response options, and question order. Based on feedback from these efforts, our team finalized the survey and programmed it into the electronic survey tablet system. Field researchers collected initial surveys (during the first 22 days of implementation) on paper and then transferred these into the tablet system to ensure its strong functioning. Subsequently, we eliminated paper surveys, and we plan to collect all remaining survey data on the programed electronic tablets, which use CommCare software.

#### Field researcher training and monitoring

All field research staff were Masters level professionals with degrees in social work or counseling and were trained on and experienced with rural field research and survey data collection in the fields of health and social services. Scientists within our investigator team select staff via a competitive hiring process, and then train them over the course of 2 weeks on family planning and CHARM2 research protocols. We also trained the field research team over 1 week on survey implementation of the CHARM2 survey, inclusive of use of the CommCare tablet system with a training manual developed for this study. An ICMR-NIRRH affiliated gynecologist provides training to the field staff on how to administer and read a pregnancy test. The scientist supervisor on site, who has more than 30 years of experience working on rural field research related to family planning, meets with the team weekly to review work, identify issues and address them, and provide supplemental training or course correction as needed. A field manager also works to oversee all field research at sites, via periodic observations, and reviews output daily to ensure quality control of data. This includes monitoring of daily enrollment data, tracking follow-up assessments and confirming a plan of action for follow-up, and monitoring the participation and follow-up rate daily to maintain high retention rates. This system enables our team to generate reports to track follow-up data on a timely basis, to identify missing or late data, and to support research staff to address any issues quickly.

#### Data management and monitoring

Field researchers upload all electronic survey data daily into the CommCare system, which allows for access to data for all investigators across institutions simultaneously, but is password protected to ensure privacy of data. All data are de-identified; our team labels electronic survey data with a unique identifier specific to an individual participant to allow for linkage of baseline and follow-up data and linking of couple data to each other, for purposes of analysis. Field research staff maintain tracking forms on paper for all participants, with identifiable information on name and location and basic demographics (e.g., age, sex), as well as additional contacts to facilitate follow-up and tracking. We store all paper-based identifying information in a locked cabinet at the field investigator site, and we maintain electronic identifiable data records linking names/locations with the unique identifier, separate from survey data, on a password-protected server that is only accessible by field staff and supervisors, to ensure quality tracking and linkage of data. We also maintain an excel monitoring spreadsheet summarizing information on schedule of study assessments and intervention delivery to ensure timely follow-up; this monitoring sheet is developed and managed by the field supervisor and used to update study investigators on data collection via twice monthly to weekly calls with the full investigator team, across India and the US. We will also use the data on intervention participation recorded in the monitoring spreadsheet for our dose analyses. To ensure high-quality survey data, our data analyst also conducts data consistency checks and provides a monthly report on these data for review by senior investigators on the study.

#### Sample size determination

At baseline *n* = 1200 sample will be collected, where 80% of couples are expected to be retained (i.e., *n* = 960) by 18 months follow up. This sample size was determined based on expected treatment effects detectable with 80% power given the 2-arm design with 20 clusters of 48 measurable couples (assuming 80% retention) per arm. Power calculations account for both within-village and within-subcenter variance in clusters.

- Based on the original CHARM study [14], we expect the proportion of women using modern contraception in the control group to be 40% at 18-month follow-up. This study will have 83.3% power to detect an absolute difference of 12% or more between treatment groups at follow-up, or 40% vs 52% in the two groups, with total kappa [variance] = 0.10, assumed to be split equally between within-village variance and within-cluster variance.
- Based on the original CHARM study [14], we expect that 89.2% of women in the control group will report no recent MSV at 18-month follow-up. We have 91.5% power to detect an absolute difference of 6% or more between treatment groups (89.2% vs. 95.2%) with kappa = 0.036, assumed to be split equally between within-village variance and within-cluster variance. The effect size of 6% difference and the kappa = 0.036 were observed in the CHARM study [14].
- Based on the original CHARM study [14], we expect the proportion of women reporting no unintended pregnancy over 18-month follow-up to be 90% in the control group. This study will have 89.5% power to detect an absolute difference of 5.5% or more between groups (90% vs. 95.5%), with kappa = 0. The effect size of 5.5% difference is based on a meta-analysis of cluster RCTs evaluating the impact of FP interventions on unintended pregnancy (with comparable measures to this study) [35]. The study showed a 50% reduced risk for unintended pregnancy amongst intervention participants, or 5.5%. The kappa = 0 is based on the CHARM study, where no significant intra-cluster correlation was found for this outcome.

While we will use longitudinal mixed-effects logistic regression methods in our analyses, for power calculations we considered only comparison of the effects at 18-month follow-up. Power calculations for each hypothesis used statistical simulations with 10,000 iterations. We converted our kappas to the random cluster effects using the delta method, and confirmed results through the simulation.

#### Data analyses

Analyses will test whether CHARM2 participants relative to control participants are:

- More likely to use contraception at 9 & 18-month follow-ups;
- Less likely to report MSV at 9 & 18-month follow-ups;
- Less likely to have an unintended pregnancy over the 9 & 18-month follow-up periods.

The primary comparison of the binary outcomes between intervention groups at 9 & 18-months will use mixed-effects longitudinal logistic regression, with random effects for the individual (couple) and for geographical clusters (SCs), over time. The baseline will be included as a time point (contraception and MSV outcomes only). A categorical time effect will be used (profile model). A difference-in-differences analysis will be used to compare the treatment arms with control, testing for an interaction between time and treatment arm in the longitudinal model. In addition, the intervention groups will be compared via a generalized estimating equations (GEE) logistic regression analysis. The GEE models will use a nested exchangeable working correlation matrix that takes into account geographic clusters and the individual (couple) within the cluster. No demographic covariates will be included in the primary analysis. Since the treatment assignment is at random, this “unadjusted” analysis will provide a causal effect of the intervention. However, we will also conduct secondary analyses, adjusting for potentially relevant covariates (e.g., age, current pregnancy, past year childbirth, etc.), in addition to time and treatment arm, using backward model selection at the α = 0.15 threshold level.

The primary analyses will use an intent-to-treat approach and analyze all subjects according to randomized group. For subjects missing month 9 or 18 endpoints, we will impute data from the lower (worse) half of the distribution by arm (conservative approach) for sensitivity analyses. We will also conduct dose analyses, in which we will include only those subjects who complete the full intervention. Analyses will use women’s data only for outcomes; however, we will use men’s data to validate outcomes a) increased contraceptive use, and c) reduced unintended pregnancy. We will conduct exploratory analyses using outcomes of contraception by type if numbers are sufficient across time points.

#### Qualitative data on outcomes

We will also collect qualitative data from a subsample of intervention participants (*n* = 50 couples, 5 couples from each of the 10 intervention clusters), to understand the significant behavior changes related to outcomes demonstrated as significant in survey data. We describe further detail on the content and procedures related to the collection of these data below, in the implementation science section, as the purpose of these interviews is also to describe implementation.

#### Cost-effectiveness

Should CHARM2 demonstrate effectiveness in accordance with our hypotheses, we will conduct a cost-effectiveness analysis using WHO-CHOICE methodology [36], an internationally accepted standard of economic analysis of health programs. A senior level economist will oversee this work. We are collecting costs of the intervention prospectively, including capital costs (e.g., equipment, space) and recurrent inputs (e.g., FP supplies, administrative costs). Given an expected outcome of improved contraception, we will include increases in non-CHARM2 FP services as partner costs. We will discount costs at a rate of 3% per year, per WHO-CHOICE recommendations, varying from 0 to 6% in the sensitivity analysis. We will assess drivers of variation in the total program cost using multivariate linear regression. We will calculate cost-effectiveness by dividing the total program costs by total health outcomes achieved over intervention duration. Our outcome measures will include number reached by the CHARM2 and unintended births averted (based on outcome analyses). Based on prior research [37], we will use an estimate of 3.8 DALYs averted per unintended birth averted. We will also include quantitative measurements of other benefits from unintended pregnancy prevention (e.g., household savings and assets) to capture some of the spillover effects of when unintended pregnancies are prevented. Following WHO benchmarks, the intervention will be categorized as ‘very cost-effective’ if the cost per DALY averted is <1x the country’s per capita GDP [38, 39]. We will undertake a probabilistic sensitivity analysis, sampling each cost and outcome from an appropriate distribution to determine mean incremental cost-effectiveness, and assess drivers of uncertainty using ANCOVA.

### Implementation evaluation of CHARM2

In addition to a rigorous quantitative outcome evaluation, this study also includes an implementation evaluation, with a number of activities to help ensure fidelity to design of the intervention and quality delivery, as well as to track and monitor elements of implementation required for replication and scalability. This component will involve quality assurance checks and monitoring using the following efforts:

1. Standard training for all providers delivering or supporting CHARM2 and training evaluation;
2. Periodic observation of CHARM2 providers by a senior researcher with the team, and provision of immediate feedback to providers;
3. Quarterly meetings among CHARM2 providers with a senior researcher with the team, to discuss difficult situations and determine ways to rectify issues arising from program implementation;
4. In-depth interviews with CHARM2 providers and select study participants to provide feedback on their view of the intervention;
5. Brief participant satisfaction surveys with all participants at the 9-month follow-up survey and with providers quarterly, to assess their response to program; these surveys will also allow us to check for contamination between treatment groups.

We have outlined details on these efforts and their purpose in Table 4. This includes information about the data forms we will use for monitoring of implementation, as well as plans for management and use of these data for quality control, to provide pragmatic feedback for the program staff, and to document implementation processes. Our implementation protocol is consistent with best practices for process evaluation and implementation science recommended by the NIH Behavior Change Consortium [40]. In addition to these efforts, we also take pictures of trainings and intervention implementation in the field, with signed authorization on photo release forms, to help document the project.

**Table 4:** Implementation Evaluation Forms and Activities for CHARM2

Form * form is data entered	Who completes form and when	Data management and when	Purpose of form	How are data reviewed and used
Screening, Recruitment, & Monitoring Sessions				
Recruitment and tracking log	Who: Field staff When: Each time a couple is contacted. Additionally, updated on a quarterly basis for as long as participants stay followed up.	Who: Field staff to collect data and enter into tables. They will review the data weekly to ensure recruitment numbers are reaching their goal, and recruitment rates are acceptable. PC will compile tables from field staff team leaders into the master excel file. When: During recruitment, weekly statistics on recruitment numbers and % of contacted couples recruited summarized. During follow-up, bi-weekly reports of contact attempts, contacts made and the current follow-up %.	To track recruitment rates, refusal rates and reasons for refusal. It will include detailed tracking information with contacts of in-laws, natal family, a neighbor on CommCare. Will also track the completion of each survey. For any loss to follow-up will track any known reason for loss to follow-up.	Reviewed by NIRRH/ PC weekly. Discussed with UCSD weekly, for recruitment numbers, and recruitment rates.
Training protocol (for intervention)	N/A	N/A	Training protocol for providers. Includes role play and scoring on role play.	N/A
Training evaluation for providers	Who: Providers When: Before and after each training, and booster training.	Who: Field staff to review forms within 30 days of the training. Could be paper-based then manually entered.	Filled pre- and post each provider training. Evaluating trainee’s knowledge before and after training to assess knowledge gained and any need for specific content in booster training. Roleplay scores can also be used for assessing need for booster training. Booster training to be conducted annually.	India team to assess topic areas that training is not effectively conveying and/or areas that need booster training. Booster and additional trainings created based on findings and need.
Monitoring Intervention Quality and Delivery				
Intervention curriculum and delivery protocol	UCSD	N/A	Intervention curriculum for 3 sessions	N/A
Session Observation/Checklist forms × 3 for each session Note: This will only take place for 10% of participants; there should be different forms for each intervention session	Who: 10% CHARM Intervention Participants only (both male and female participants) When: At the end of each randomly selected session	Who: Field staff will collect and give to field manager to review and process data When: Once a month. Casefile analyzed as needed bases or at time of observation.	Quality assurance to ensure adherence to curriculum and that providers are providing the full program to participants	Reviewed by NIRRH and PC program manager, monthly; Feedback provided to Providers in monthly meetings. Entered with quantitative data; analyzed biannually; prevalence data used for funder reports
Participant and Provider Feedback on Intervention				
Participant Satisfaction Survey	Who: Research team administers satisfaction survey to participants. When: With the 9 months, or 18 months follow up survey if not available at 9-month follow-up	Who: Field staff will collect data. The field manager will input data. Program manager will manage the data When: Frequencies to be run by data analyst monthly to review quality and fidelity data.	Participant satisfaction with CHARM2 intervention	Discussed quarterly with UCSD. India team to discuss in their meetings.
IDIs with couple participants (husband and wife separately)	Who: Research team administers satisfaction survey to 10% of participants who received intervention and 10% of participants who quit When: 9 months follow up	Who: Data are translated and transcribed by the field team in India. When: Within 2 days of interview completion.	To document participants’ perceptions of what they received in the intervention, what they learned from the provider, the quality of that learning and how that learning affected their relationship, FP and MSV.	Discussed twice a year. UCSD will compile data and select quotes for inclusion in reports.
IDI with providers	Who: Research team administers satisfaction survey to participants When: Biannually, when no survey data is being collected	Who: Field research team When: Twice a year, 6 months apart.	To document providers experiences with intervention delivery, their perceptions of how the intervention affected couples and how they continue to see these couples	Discussed twice a year. UCSD will compile data and select quotes for inclusion in reports.

Note: The field manager and the scientist overseeing the field team will maintain notes from their ongoing meetings with field staff and from observations of intervention delivery, to discuss key issues and generate plans for course correction or additional trainings as needed during the weekly to twice monthly meetings with the full scientific investigator team

All above data collected from providers and participants are for purposes of implementation evaluation only, and we will collect these data only after receipt of informed written consent. We will use simple descriptive analyses to analyze all quantitative data collected for these purposes. Qualitative data will be formally analyzed.

#### Qualitative data for implementation evaluation

We will collect in-depth interview data from a subsample of CHARM2 participants and all CHARM2 providers to provide insight into the process of intervention delivery, how it affects behavior and norm changes, and how the program is perceived and valued. A subsample of CHARM2 couples (*n* = 50, 5 couples per intervention cluster) will be recruited to participate in in-depth interviews at 9-month follow-up to provide insight into their perceptions of the CHARM2 intervention and its value, as well as their experiences with FP and in their marital relationship, generally and as a consequence of CHARM2 participation. We will conduct interviews separately for women and men, with a gender-matched interviewer. We will attempt to include couples who received the full intervention and those who received only part of the intervention. We will also conduct in-depth interviews with CHARM2 providers (minimum *n* = 20) to explore providers’ perspectives and experiences delivering CHARM2 and to assess their perceived impact on couples. In-depth interviews with the providers will explore their experience in delivering intervention sessions, both individual and couples’ sessions, areas for improvement, community acceptance, and perceived sustainability of the program. While couple interviews will occur one time, at 9-month follow-up, providers actively delivering the intervention will be asked to participate in these interviews twice a year, or every 6 months to assess ongoing intervention delivery. No incentives are offered.

Our trained research team will conduct all interviews in a private location, with a gender-matched researcher. We will record the interview unless the participant refuses, in which case, the field researcher will take detailed notes in English. Subsequent to the interview, and within 48 h, the researcher will transcribe/translate these interviews simultaneously for analysis. We will maintain de-identified digital recordings and transcriptions, labelled with the participant’s unique identifier, to link qualitative and quantitative data and to link provider and participant data. We will import transcribed texts into ATLAS.ti®, software that helps to organize and facilitate analysis of qualitative data [41]. We plan to analyze these data using a grounded theory approach, in which there is a continuous interplay between data collection and analysis to iteratively generate themes and adapt interview guides [42–44]. We will use the following approach to our analysis:

1. Data Immersion- The field team and senior scientist investigator on site will read and reread transcripts and associated field notes to familiarize themselves with the narratives and create memos to guide theme development;
2. Theme Generation- The field team, again under the guidance of the senior scientist in the field, will develop a list of emergent key themes seen across narratives;
3. Framework Development- Our scientific investigator team will review and discuss the generated themes and their intersections, to create a framework to understand the issues of focus. We may develop revisions to protocols or extensions of data collection to better gain more insight;
4. Codebook Development- The senior scientist investigator will develop a codebook based on the framework themes, and a coding structure will be created to guide further data analysis;
5. Coding and Iterative Analysis- The field team will code and analyze all data using themes generated in steps 2–4. We will have two coders review and code each transcript using Atlas-ti. Coders will code separately, and codes will be analyzed for inter-coder reliability based on Cohen’s Kappa [45]. We will review coding discrepancies to reach consensus. New codes may emerge, and we may expand the codebook and conduct another analysis of the data. We may also recruit additional participants to reach saturation in theme generation.

Based on findings from this approach, the framework will be refined with the goal of helping clarify how the intervention is able to support couples, and how the providers effectively deliver the intervention.

### Data safety and monitoring and protections for human subjects

For all aspects of this study, we have developed our protocols to maintain ethical treatment of study participants in accordance with our guidelines from our institutional review boards. With regard to data safety monitoring of study participants, we focus on two major safety aspects: 1) assurance that no harm comes to participants as a result of survey or program participation and 2) assurance that all data collected from this project maintain the privacy of research participants. Our senior scientists based in India and included as co-authors on this paper monitor the work in the field to ensure we adhere to our monitoring protocols, with a designated site Principal Investigator providing oversight.

To ensure that no harm comes to our participants, we have instituted a number of protections. All participation in this study requires written informed consent, ensuring that all participants understand the nature of the study, our efforts to maintain confidentiality, and their right to withdraw from the study at any point. The informed consent process also includes the name of an India-based senior scientist overseeing ethical treatment of participants, as noted above; we advise all participants that they may contact this individual if they have any concerns related to the study. We also make every effort to minimize participants’ risk of a loss of confidentiality during this study. The research team will not inform participants’ family members or others of their participation, and we will not inform any spouse of the other’s assessments as part of this study. We will de-identify all data. We have also asked all research staff and providers to maintain the confidentiality of all participants in the study. As noted above in our description of data management, we will keep electronic files only in a password protected project drive, and we will maintain paper forms from the study in a locked file. All electronic files will be backed up nightly to minimize the likelihood of lost files or data.

Given the high rates of spousal violence seen in India and globally, we have also included certain procedures to reduce vulnerability for women experiencing spousal violence, based on the WHO guidelines for domestic violence research [46], and using an approach we have used previously in India with no issues [14], as follows (and noted in our procedures above):

- If women at any point in the study report life-threatening violence from husbands or in-laws, these women will be withdrawn from the study and provided with supported referral (assistance with transport and accompaniment) to the Mahila Hakka Suraksha Samiti, a local domestic violence agency by research staff. If non-life-threatening violence from husbands or in-laws is reported, we will also share information about local domestic violence services with the participant.
- All research staff and intervention providers will be trained on how to work with women affected by domestic violence [46]. For example, this includes using “dummy” questions if privacy is breached during an interview or an ANM provider visit, and training staff to identify when women are distressed and need debriefing or immediate health attendance. They will also offer support to conduct a safety plan with a woman reporting spousal violence to ensure she has thought through ways to safely escape her situation should she choose to do so.
- Research and program staff will never disclose to any family member who participated in the study, what questions were asked of them, or what responses were given. Participants will also be asked not to share the questions and their responses with others, particularly if they think it could put them at risk for abuse.
- We will assess women for domestic violence, in private places or environments where we can assure confidentiality. As part of informed consent, we clarify to all participants that they may refuse response to a question, including questions on spousal violence.
- We train research staff to debrief with participants as needed, validating that violence is never the victim’s fault, there are services to support women in this type of situation, and that the information provided will not be shared with others in an identifiable format.

Additionally, we have developed both internal and external data safety and monitoring procedures, and a plan to determine if halting of study procedures are required due to an identified harm to participants or others engaged in the study. While we do not anticipate that study involvement will cause harm, if this does occur, we will adhere to the strict reporting requirements for adverse events required by our institutional review board approvals, inclusive of an annual reporting process.

The research team, in India and the U.S., will undertake internal data safety and monitoring, as described throughout this paper, and under the direction of the Principal Investigator of the study, based at the University of California San Diego. As noted previously, we hold scientific investigator meetings weekly to twice a month to review and monitor data and field activities and address any identified concerns. However, if the field team identifies an adverse event, they will notify the Principal Investigator by email within 72 h, and if it is a serious adverse event, they will notify her within 24 h. She, and all investigators overseeing IRB protocols related to this study, will inform their IRB within 48 h of receiving notification of an adverse event or serious adverse event. Meetings of the scientific investigations will also be set within this period to consider how to address the concern and reduce or eliminate the identified risk, if related to the project and under our control.

We also have a Data Safety and Monitoring Board (DSMB), developed for this study as an external monitoring body. Our DSMB consists of three independent scientists with whom we meet via phone annually to review the study with them. We have asked this board to review all procedures related to the protection of study participants, including confidentiality procedures and reports of distress. None of these scientists are otherwise involved in this study, allowing objectivity regarding how best to handle an identified adverse event. If we identify an adverse event, we will also review it with our DSMB to obtain input on how to address the concerns. We will only bring an issue to the attention of the DSMB if one of the study investigators identifies a need within 72 h of the standing meeting, at which point that investigator will inform the team of his/her intention to bring this forward. Otherwise, we will update the DSMB twice a year on all identified adverse events, to provide them with opportunity for input, unless no adverse events have occurred. If such updates are required, we will draft a report reviewing all adverse events or unexpected problems to date to share with our DSMB and IRBs.

We do not anticipate any serious adverse events from this study, but if we feel that the study is placing women at increased risk for violence from their families or develop any concerns, and we feel that we cannot rapidly alter the study to reduce or eliminate this risk, we will immediately halt the study.

#### Ethics approvals

All partner institutions on this study, the University of California San Diego, the National Institute for Research in Reproductive Health in India, and the Population Council, have obtained approval from their respective institutional review board for this study.

## Discussion

The CHARM2 Intervention is a five-session, gender-synchronized intervention involving gender equity and family planning counseling for married couples in rural Maharashtra, India. The CHARM2 intervention builds upon a previous iteration of the CHARM model which focused on male-sessions, but not female sessions, to support gender equity and family planning [13]. While the original CHARM intervention demonstrated increased modern contraceptive use, it showed no impact on unintended pregnancy, likely due to reliance on lower effectiveness forms of contraception such as male condoms [14]. CHARM did demonstrate reduced risk for MSV, a concern for almost 1/3 of women in the study sample [14] and a significant risk factor for condom non-use and oral contraceptive failure [15, 16]. These findings indicated that original CHARM sessions for men would benefit from sessions for women, ideally delivered by a female ANM in the public health system and inclusive of a broader array of contraceptive options. We designed CHARM2 to utilize this gender-synchronized approach.

This protocol details planned evaluation of the CHARM2 intervention to assess its impact on reversible contraceptive use, MSV, and unintended pregnancy. The evaluation includes both an outcome evaluation and an implementation evaluation to assess quality of delivery and document implementation for replicability and scale-up. We will conduct our outcome evaluation via a two-arm cluster RCT, which compares CHARM2 participants with the control arm, which receives SOC FP services from the public health system. We are administering surveys on family planning behaviors and preferences to all participants, intervention and control, and pregnancy tests to all female participants, at baseline, 9 months follow-up and 18 months follow-up. We will evaluate the impact of CHARM2, relative to the control condition, on reversible contraceptive use, pregnancy, and MSV over time.

We are also collecting qualitative data in the form of in-depth interviews from both CHARM2 participants and providers, to provide insight into how the intervention affects these outcomes as well as perceptions of the value of the intervention, the latter supporting the implementation evaluation goals. Our implementation evaluation involves a number of activities, including these qualitative data efforts, to help ensure fidelity to design of the intervention and quality delivery, as well as to track and monitor elements of implementation required for replication and scalability. Included in these activities are standardized training and monitoring of CHARM2 providers and participant satisfaction surveys from all participants, allowing for comparisons of exposure and experiences with family planning counseling across treatment groups. Should CHARM2 prove effective and sustainable, study findings will have broad implications for FP interventions for low resource settings in India and elsewhere. Additionally, curricula, training protocols, evaluation protocols, cost effectiveness findings will have been developed to support its implementation and evaluation with other populations.

## Data Availability

Not applicable.

## Abbreviations

ANM: Auxiliary Nurse Midwife
ASHA: Accredited Social Health Activist
BCT: Balanced Counseling Strategy
CHARM: Counseling Husbands to Achieve Reproductive Health and Marital Equity
Co-I: Co-Investigator
DHS: Demographic and Health Survey
GAD: Generalized Anxiety Disorder Scale
GEH: UC San Diego’s Center on Gender Equity and Health
IDI: In-Depth Interview
IQFP: Interpersonal Quality of Family Planning scale
IUD: Intrauterine Device
LARC: Long-Acting Reversible Contraception
MSV: Marital Sexual Violence
NFHS: National Family Health Survey
NIRRH: National Institute for Research in Reproductive Health (India)
PC: Population Council
PHC: Primary Health Center
PHQ: Patient Health Questionnaire
PI: Principal Investigator
RCT: Randomized Control Trial
SC: Subcenter
SCT: Social Cognitive Theory
SOC: Standard of Care
TGP: Theory of Gender and Power
